# Response: Commentary: Distrust, False Cues, and Below-Chance Deception Detection Accuracy: Commentary on Stel et al. (2020) and Further Reflections on (Un)Conscious Lie Detection From the Perspective of Truth-Default Theory

**DOI:** 10.3389/fpsyg.2021.763218

**Published:** 2021-10-21

**Authors:** Mariëlle Stel, Eric van Dijk

**Affiliations:** ^1^Department of Psychology of Conflict, Risk, and Safety, University of Twente, Enschede, Netherlands; ^2^Department of Social, Economic, and Organisational Psychology, Leiden University, Leiden, Netherlands

**Keywords:** deception, distrust, deception cues, lie detection, consciousness

## Introduction

People often mistake other people's deceits for truths (i.e., the truth bias; McCornack and Parks, 1986). The Adaptive-Lie-Detector theory suggests that people make informed judgments using reliable cues. A possible explanation for the truth bias is that when cues are absent, people make an “educated guess” based on most communication being honest (Street, 2015). Stel et al. (2020) investigated whether inducing contextual distrust could be the antidote for this bias. Based on previous evidence that (1) distrust may induce conscious thought (e.g., Mayo, 2015) and (2) conscious processes can hinder the ability to detect deception (e.g., Reinhard et al., 2013), we expected and found that participants are less accurate in judging deceits and truths when contextual distrust (vs. trust) is induced, which was partly due to participants relying more on false beliefs about deception.

In his commentary on Stel et al. (2020), Levine (2021) agreed that (1) distrust hampers correct deception judgments and that (2) distrust involves conscious processing. He was, however, skeptical that deception cues could explain why distrust hampered truth detection. The main arguments were that Stel et al. found (1) below chance-accuracy in the distrust condition, (2) which was explained by more reliance on false deception cues. Levine states that the deception cues used in Stel et al. are generally non-diagnostic rather than antidiagnostic. He argued the findings are not in line with previous findings and his theoretical perspective. Here, we react to these comments and argue that our findings do not contradict, but expand previous findings.

## Below-Chance Accuracy

First, Levine challenged the below-chance accuracy in the distrust condition, mentioning that with conventional research designs, people show above-chance accuracy overall (Bond and DePaulo, 2006; Levine, 2020). The studies cited by Bond and DePaulo and also Levine, however, investigated detecting deception without distrust induction. The studies of Stel et al. (2020) involved inducing contextual (dis)trust and therefore cannot be one-on-one compared with studies in which no induction was involved. Also note that accuracy rates can vary dependent on context and that below-chance accuracy has been found in previous research (Levine et al., 2005).

Furthermore, Levine (2021) mentioned that while suspicion or distrust decreases accuracy for truths and increases accuracy for lies, this would not explain elimination of the veracity effect as found in Stel et al. (2020). He based this on the suspicion research of Kim and Levine (2011). First, we like to point out that it is important to take all studies on suspicion into account, including studies that found different results (Zuckerman et al., 1982; Burgoon et al., 1994; Levine et al., 1999) or little or no effect (Toris and DePaulo, 1985; Buller et al., 1991; Stiff et al., 1992). More importantly these studies address suspicion—not distrust. The two concepts are distinct (Sinaceur, 2010): Suspicious people are uncertain about other people's motives, but distrusting people have negative expectations about these motives. The level of suspicion in Stel et al. was constant across (dis)trust conditions: All participants knew beforehand that they would rate targets' truthfulness and were moderately uncertain about the targets' motives. In addition, we subtly manipulated (dis)trust by having participants adopt facial expressions in line with either contextual distrust (eye-squinting) or trust (eye-rounding).

These effects of contextual (dis)trust on deception detection cannot be equated to either (1) studies in which no distrust was induced or (2) studies on suspicion only. Our findings that contextual distrust produced below-chance accuracy therefore do not contradict, but supplement previous research.

## Cues as Mediating Mechanism

Levine (2021) also mentioned that the proposed mechanism of Stel et al. (2020) lacks plausibility as the false beliefs that partially mediated the effects in Stel et al. would generally be non-diagnostic and could therefore not lead to below-chance accuracy. First, because the false belief cues of our paper included the antidiagnostic cue more hand movements (DePaulo et al., 2003; Sporer and Schwandt, 2007) it is conceivable that relying on the false belief cues leads to poorer deception detection (hand movements was mentioned by 27.8% of all participants). This is also in line with Vrij et al. (2001).

Furthermore, we obtained *partial* mediation, suggesting there may be another mechanism at play, which we did not measure. As mentioned in Stel et al. (2020), it is possible that participants also relied on other cues which they did not report (Hartwig and Bond, 2011). That we did not find diagnostic cues to facilitate detecting deception may be due to using non-verbal deceit and truths only, again as explained in Stel et al. The limited number of diagnostic cues could also explain why we did not obtain a (partial) mediation of diagnostic cues; generally multiple cues increase diagnostic value (Hartwig and Bond, 2014).

## Discussion

In sum, even though below-chance accuracy is less common in deception research, our findings are the result of investigating a new contextual effect in which distrust was induced. This induction, on top of participants' moderate suspicion, may have led to an even stronger decrease of the truth-default. Furthermore, the non-verbal context and the inclusion of an antidiagnostic cue may have led to a partial mediation of false deception cues.

We realize that some deception researchers are skeptical about research focusing on deception cues as these have proven to be weakly related to deception (Hartwig and Bond, 2011). However, the state-of-the-art also includes research that does show the involvement of deception cues in deception detection (e.g., Vrij et al., 2001; Reinhard et al., 2013), which cannot be ignored. It is conceivable that there may be undiscovered moderators at play. Furthermore, meta-analytical databases on deception cues need to be updated with newer studies with high coding reliability (Sporer and Ulatowska, 2021). It is therefore important to share all findings—even the findings that at first sight do not seem to fit previous research—as it helps us to further theorize and understand how and when deception cues influence people's deception detection abilities. The results of Stel et al. (2020) add to the understanding that deception cues play a role in conscious information processing hindering truth detection.

## Author Contributions

MS wrote the first draft of the manuscript. ED revised the manuscript. All authors contributed to manuscript revision, read, and approved the submitted version.

## Conflict of Interest

The authors declare that the research was conducted in the absence of any commercial or financial relationships that could be construed as a potential conflict of interest.

## Publisher's Note

All claims expressed in this article are solely those of the authors and do not necessarily represent those of their affiliated organizations, or those of the publisher, the editors and the reviewers. Any product that may be evaluated in this article, or claim that may be made by its manufacturer, is not guaranteed or endorsed by the publisher.
